# Retrospective Analysis of Compulsive-like Long-Standing Unacceptable Urination in Cats

**DOI:** 10.3390/vetsci13030234

**Published:** 2026-02-28

**Authors:** Stefania Uccheddu, Federica Pirrone

**Affiliations:** 1 San Marco Veterinary Clinic and Laboratory, Via dell’Industria 3, 35030 Veggiano, Padua, Italy; 2 Department of Veterinary Medicine and Animal Sciences, University of Milan (UNIMI), Via dell’Università, 6, 26900 Lodi, Lodi, Italy; federica.pirrone@unimi.it

**Keywords:** clomipramine, retrospective study, behavioural modification

## Abstract

Unacceptable elimination is a common and frustrating feline behavioural complaint and, when expressed as a repetitive and rigid ritual, it may resemble a pattern of compulsive-like elimination. This retrospective case series reviewed medical records from a single veterinary behaviour service (July 2020–July 2025) to describe cats with longstanding unacceptable urination showing caregiver-reported compulsive features (repetitiveness, redundancy, and rigidity) and to compare outcomes between behavioural modification alone (group B) and behavioural modification plus clomipramine (group C). Twenty-one cats met the inclusion criteria (C, *n* = 11; B, *n* = 10) after relevant medical causes had been excluded via physical examination and laboratory testing. Baseline characteristics were comparable between groups, including age, sex distribution, and interval between onset of clinical signs and initiation of therapy. Clinical outcome and time to recovery differed markedly: a positive outcome was recorded in 1/10 cats in group B versus 10/11 in group C after 30 days of therapy (first follow-up). Follow-up completion was also consistently higher in group C across all time points. These findings might suggest a pattern of compulsive-like elimination, with the observed response to clomipramine providing further support for this conceptual framework.

## Simple Summary

Unacceptable urination is a common reason for caregivers to seek help and, in severe cases, to give up the cat. In some cats, the behaviour becomes very repetitive, happening in the same way and in the same places every day, even when the original trigger has been removed. This study reviewed clinical records of 21 cats that had been urinating outside the litter box for at least one year and whose caregivers described the behaviour as rigid and repetitive. After ruling out medical causes with examinations and laboratory tests, cats were managed either with behavioural advice alone or with behavioural advice plus clomipramine, a medication commonly used for anxiety and compulsive-type behaviours. Cats receiving clomipramine improved more often and much faster than cats receiving behavioural advice alone. All cats in the clomipramine group achieved a positive outcome within one year, while only a minority in the behaviour-only group did. Cats treated with clomipramine also reached recovery sooner and caregivers were more likely to complete follow-up checks over one year. These results suggest that when unacceptable urination shows compulsive-like features, it may be more appropriately conceptualised and managed as a compulsive behaviour, with combined pharmacological and behavioural treatment leading to faster clinical resolution.

## 1. Introduction

Feline behaviour problems represent a substantial proportion of cases referred to behavioural medicine specialists, with chronic anxiety disorders being particularly prevalent [1]. These disorders often stem from an individual cat’s maladaptation to environmental stressors, such as the presence of conspecifics or humans, exposure to noise, or prolonged social isolation. Treatment typically involves a multimodal strategy that combines environmental and behavioural modification with pharmacological interventions, including the use of anxiolytic agents such as clomipramine or fluoxetine, both of which have consistently proven effective [2]. In domestic cats, house-soiling disorders are among the most frequent behavioural complaints and a leading cause of relinquishment [3,4]. Following the diagnostic categorization of house-soiling cases, the literature identifies the following primary aetiologies [5,6]: (1) Medical conditions, including urolithiasis, chronic kidney disease, urinary tract infections, diabetes mellitus, hyperthyroidism, and orthopaedic or neoplastic pathologies; (2) Feline Idiopathic Cystitis (FIC); (3) Urine marking behaviour; (4) Elimination issues related to environmental or social stressors. The specific aetiology of unacceptable elimination in domestic cats is frequently multifactorial, stemming from a complex interplay of environmental and psychosocial stressors. In multi-species households, a primary trigger is the perceived lack of security at designated latrine sites. Social tension, whether intra-species or caregiver-related, can significantly diminish feline self-confidence, resulting in the compensatory selection of ‘‘protected’’ or elevated locations that offer a heightened sense of safety.

Consequently, the clinical management of such cases necessitates a comprehensive understanding of ethologically normal feline toileting patterns. It requires identifying the specific sensory or environmental drivers that render a cat’s preferred alternative location or substrate more attractive than the facilities provided by the owner. Ultimately, this behavioral shift may represent a functional adaptation to an environment that fails to fulfill species-specific requirements for privacy and secure access to resource.

While elimination may serve a communicative function, especially in marking behaviour [7], when it becomes rigid, repetitive, and functionally dissociated from its original purpose, it may shift from a communicative or anxiety-related response to an unacceptable and compulsive-like behavioural pattern [8]. However, it is crucial to consider that repetitive behaviours in cats may emerge as displacement activities or stress-coping mechanisms triggered by frustration and fear, rather than representing a primary Compulsive Disorder (CD) [9]. According to the psychobiological approach [10], a significant link exists between discrete emotions—such as frustration or fear—and the expression of repetitive elimination outside the litter box. These responses are modulated by the subjective salience of stimuli within both the internal and external environments. Consequently, emotional responses are considered discrete insofar as a specific emotion organizes a repertoire of actions toward a common functional goal. Redundancy and rigidity—two phylogenetically conserved features underlying the stereotypic motor patterns of repetitive behaviours in both humans and other animals [11]—form the basis of ritualisations. In clinical practice, such ritualisations, typically marked by a loss of the original behaviour’s function and a fixation on the precise execution of acts, offer a conceptual bridge for interpreting behaviours that may retain an apparently functional purpose yet become problematic when exaggerated, displayed out of context, or are disruptive to daily activities [12]. Beyond the persistence and ritualised nature of unacceptable urination and its disruptive impact on quality of life, responsiveness to anti-compulsive pharmacotherapy suggests that feline unacceptable elimination may be conceptualised as a pattern of compulsive-like elimination [13]. This perspective reframes a common and often frustrating behavioural problem while also opening new avenues for research and treatment and clarifying the continuum between normal elimination, anxiety-related elimination, and pathological elimination. By distinguishing functional from structural aspects of elimination, this perspective helps clarify how normal and anxiety-related behaviours may progress into compulsive patterns, thereby improving diagnostic accuracy and supporting more targeted interventions.

To investigate the potential pattern of compulsive-like behaviour of feline unacceptable elimination, we retrospectively reviewed clinical records and selected cases based on the presence of core behavioural features consistent with compulsive like patterns, namely repetitiveness, redundancy, and rigidity, as reported by owners. In addition, because compulsive behaviours typically emerge in situations of frustration or conflict but persist beyond the original eliciting context, only cases in which identifiable environmental or social triggers had been previously addressed as part of routine clinical management were included. Following trigger management, cats had received either behavioural modification alone or a combined behavioural and pharmacological intervention, as documented in the clinical records. In the combined-treatment group, pharmacological management consisted of clomipramine, a tricyclic antidepressant (TCA) with demonstrated clinical efficacy in the management of urine-related behavioural problems in cats [14]. Clomipramine primarily acts by inhibiting the reuptake of serotonin and norepinephrine and exhibits additional anticholinergic, antihistaminic, and α_1_-adrenergic antagonist properties [15].

Clomipramine has proven efficacy in treating behaviours associated with both anxiety and obsessive–compulsive disorders, frequently at the core of unacceptable urination [16].

Pharmacological treatment is often deferred because of caregiver reluctance, which may contribute to therapeutic delay [17,18]. In CD, postponing medication can be associated with a reduced likelihood of improvement and prolonged patient distress, potentially leading to caregiver frustration, reduced adherence, and premature discontinuation of follow-up [17,18]. Notably, clomipramine is frequently prescribed in routine practice with an ‘anxiety’ rationale [19]. Whilst anxiety may indeed contribute, a careful behavioural assessment suggests that the clinical picture often extends beyond anxiety alone and includes a prominent CD-like component [9]. Recognising this broader framework may support earlier, evidence-based intervention and more effective communication regarding the rationale for pharmacotherapy when an CD-like phenotype is suspected. Based on our clinical experience, over 50% of these cases exhibit patterns characterized by rigidity, repetitiveness, and redundancy. However, these indicators are frequently overlooked during routine history-taking. We believe it is essential to incorporate these specific questions into consultations—not to over-diagnose compulsive disorders, but to ensure that cases presenting with these traits receive the most appropriate intervention.

Our working hypothesis is that, in cases of cats displaying unacceptable urination, behavioural treatment alone may be insufficient, as compulsive-like components are often less responsive to behavioural modification in the absence of pharmacological support. By the time these CD cases are assessed, the behaviour may already have become generalised and relatively independent of the original eliciting conditions, which contributes to its resistance to behavioural intervention alone. Given the exploratory and descriptive nature of this retrospective study, which aims to generate hypotheses for future research, the primary objective is investigate long-standing cases of house-soiling to determine if they share clinical features with CD.

## 2. Materials and Methods

### 2.1. Study Design

This retrospective, observational descriptive study reviewed medical records and owner-supplied video recordings from the San Marco Veterinary Clinic between July 2020 and July 2025. All caregivers had previously provided written informed consent for the use of their animals’ clinical data for research purposes. Cats were classified into two groups according to the treatment they had been implemented during clinical practice:

Clomipramine group (C)—cats receiving clomipramine (Clomicalm, Virbac 06516 Carros, France) (1.0 mg/kg q24 h) in association with behavioural modification.

Behaviour-only group (B)—cats receiving behavioural modification without pharmacotherapy.

The severity of cases was considered equivalent based on the daily frequency of inappropriate urination. Inclusion in the B group was reserved for families demonstrating high compliance and a specific willingness to pursue behavioural therapy as the sole intervention. Conversely, the C group comprised families who, while also meeting the essential requirement of high compliance and adherence to behavioural therapy (conditio sine qua non), agreed to proceed with a combined pharmacological approach. Both groups followed the same behavioural protocol.

Cats were eligible for inclusion if they met the following conditions:

(1) Comprehensive behavioural assessment
(2) Normal results had been obtained from a complete blood count, serum biochemistry panels, urine analysis, and ultrasound
(3) A follow-up period of at least one year after the first behavioural consultation was available in the medical record.
(4) Complete clinical records were accessible, including physical examination findings and exclusion of common infectious diseases.
(5) A documented history of at least one year of unacceptable urination was present, and caregivers described the cats’ urine deposition as a ritualised behaviour that severely interfered with normal daily functioning. All cats showed core features of repetitiveness, redundancy, and rigidity and displayed unacceptable urination, with episodes occurring daily in the same locations and manner (repetitiveness), multiple closely similar elimination events within restricted areas and time frames (redundancy), and persistent attempts to eliminate in or near their usual sites even when direct access was prevented (rigidity).

### 2.2. Diagnostic Protocol

All cats underwent a thorough physical examination was performed to rule out any underlying medical conditions that might be contributing to the cat’s observed behavioural changes. Haematological, biochemical, and urine examinations were performed for each cat. Diagnostic imaging (ultrasound) was performed when clinically indicated. This exclusionary process systematically eliminated alternative medical or environmental causes. Unacceptable urination was diagnosed only after underlying medical conditions had been excluded and behavioural counselling had been offered. Upon the initial behavioural evaluation, each cat was not receiving any behavioural therapy. The study population comprised cats exhibiting elimination issues primarily linked to environmental or psychosocial stressors. The most frequently identified contributing factors included inter-cat tension in multi-cat households, suboptimal litter box placement or management, and recent environmental changes reported by caregivers. In accordance with established clinical criteria [6,20], cases were identified based on specific ethological markers of toileting behaviour rather than marking. Specifically, inclusion required a consistent elimination pattern identified through caregiver-reported observations collected under structured clinical guidance and supported by indirect indicators consistent with elimination (e.g., squatting when observed, pooled urine on horizontal substrates indicative of full voiding, and substrate-directed behaviours such as covering. Surface orientation alone was not considered sufficient for classification. These features, namely posture, substrate interaction, and voiding quantity, are recognized diagnostic indicators to differentiate unacceptable indoor elimination from territorial marking [6,20]. To prevent misdiagnosis, cats showing any evidence consistent with urinary marking, including marking on horizontal surfaces and irrespective of territorial or emotional aetiology, were excluded.

### 2.3. Behavioural Counselling

A detailed behavioural history was collected, including the environmental context, household social dynamics, and litter box management. Key components of the behavioural consultation included direct observation of clinical signs, exclusion of underlying medical causes, and a comprehensive assessment of the cat’s emotional well-being. Caregivers were asked to provide video recordings of the urination episodes when available and a home map indicating litter box locations, sites of urination outside the litter box, resting areas, privacy areas, play areas, and feeding areas. A detailed caregiver interview provided information on the presenting complaint, the cat’s medical and behavioural history, and its interactions with household members. An emotion-focused behavioural ethogram indicated that the cat displayed fear- and anger-related responses [21]. The cat’s predominant behaviours and associated emotional states were assessed in accordance with the established behavioural literature [21,22]. The ethogram was an effective instrument for enabling the caregiver to recognise the cat’s emotional responses. During consultations, the veterinary behaviourist provided individualised behavioural guidelines, structured according to current evidence-based recommendations, following AAFP/ISFM Feline Environmental Needs Guidelines [5,23,24,25,26,27,28]. Behavioural modification strategies were structured in accordance also with the Five Pillars Framework [29]:

1. Safe space—provision of secure retreats for refuge and escape.
2. Resource availability—ensuring access to multiple, spatially separated feeding, litter, and resting areas.
3. Play and predation—opportunities to engage in hunting, climbing, and exploration.
4. Positive social interaction—fostering appropriate human–cat interactions and minimizing social stress.
5. Olfactory enrichment—respecting scent marking (rubbing and scratching) and providing olfactory stimulation.

In addition, all potential factors were systematically evaluated and addressed, including litterbox aversion, location aversion or preference, substrate preference, the role of fear, anxiety, stress, conflict, or panic within the household, underlying pathophysiological causes, and issues related to multi-cat environments.

### 2.4. Data Collection

The following variables were extracted from the clinical records:

(a) Signalment and demographic data, including breed, sex, reproductive status, and age at the time of the initial behavioural consultation and/or first prescription.

(b) Anamnestic (es. patient profile and medical history) and behavioural data, including the behavioural diagnosis(es), case history, and caregiver-reported behavioural changes.

(c) Clinical findings, including laboratory test results and any diagnostic imaging performed to exclude underlying medical conditions.

(d) Temporal variables, including duration of clinical signs, the interval between clinical onset and initiation of therapy, duration of therapy, and time to clinical recovery, defined as the number of days from therapy initiation to the absence of unacceptable urination. The clinical recovery time was documented in owner-kept diaries. It was defined as the number of days between the initiation of therapy and the first day without unacceptable urination, provided this was followed by at least one week of consecutive successful days.

(e) Clinical outcomes were defined as follow-up outcomes. Follow-up assessments were predefined a priori at 1, 3, 6, and 12 months after initiation of therapy and were collected systematically for all cases using the same clinical protocol. A follow-up time point was considered completed when at least one documented clinical update was available in the medical record within the scheduled window (e.g., caregiver email/phone update, recheck visit note, or structured questionnaire/diary entry), regardless of the clinical content (improvement or persistence of signs). If no update was available for that time point, it was classified as not completed. At each time point, follow-up was recorded as a binary categorical variable and included two components: positive/negative; coded 1/0. When a time point was completed, outcome was coded as positive (1) if the caregiver reported complete absence of unacceptable urination since the previous assessment, and negative (0) if unacceptable urination persisted or recurred during that interval. Follow-up information was supported by caregiver diaries. To systematically document behavioural patterns, caregivers maintained a structured daily diary. For each episode of unacceptable urination, owners recorded the frequency, timing, and specific locations of the events, as well as any persistent attempts to access particular sites (observable features related to rigidity, repetitiveness, and redundancy, based on established definitions in the clinical literature [30,31]. Furthermore, any deviations from the cat’s daily routine—such as the presence of visitors, changes in household composition, or exposure to loud noises—were documented to differentiate potential environmental triggers from compulsive behavioural presentations.

In addition, adverse effects, therapy adjustments, and relapses within the 12-month follow-up period were recorded.

### 2.5. Statistical Analysis

Time between onset of clinical signs and initiation of therapy were compared using the Mann–Whitney U test, as data were not normally distributed and sample sizes were small. Effect sizes for between-group differences were calculated using Hedges’ g, which adjusts for bias in small samples [32]. Time to clinical recovery was analysed using Kaplan–Meier survival analysis. Follow-up outcomes at 1, 3, 6, and 12 months were binary variables (positive vs negative outcome; coded 1/0) and were summarized as proportions and compared between groups using Fisher’s exact test, given the small sample counts. A *p*-value < 0.05 was considered statistically significant. All statistical analyses were conducted using SPSS version 28.0 (IBM SPSS Statistics for Mac, Armonk, NY, USA).

## 3. Results

A total of 21 animals were included (11 in group C and 10 in group B). Demographic details are reported in Table 1. All cats were Domestic Shorthair, except for one Siberian. No significant demographic differences were observed between Group B and Group C. The median age was 3.7 (range 2.0–5.9) and 5.0 (range 3.00–5.2) years, respectively, with a similar prevalence of male subjects (60.0% vs. 63.6%).

The proportion of males was similar (6/10, 60.0% in group B vs. 7/11, 63.6% in group C; Fisher’s exact test OR = 0.86, *p* = 1.000). All animals in both groups underwent ultrasonography (100% in both groups). The time interval between onset of clinical signs and initiation of therapy was also comparable between groups (group B: 382.5 days IQR 365.0–425.0 vs. group C: 400.0 days IQR 365.0–440.0; Mann–Whitney U = 54.0, W = 109.0, z = −0.04, *p* = 0.973).

Clinical outcome differed markedly between the two treatment groups. A positive outcome was recorded in 4/10 animals in group B and in all 11/11 animals in group C (*p* = 0.001; Fisher’s exact test). Follow-up results are reported in Table 2. A significant difference in time to clinical recovery was observed; specifically, Group C showed a significantly shorter recovery time compared to Group B (log-rank test: χ^2^ = 15.818, df = 1, *p* < 0.001).

The median time to clinical recovery was 30 days in group C, whereas the median was not reached in group B within the observation period.

## 4. Discussion

The present study compared clinical outcome, time to recovery, and follow-up between cats treated with a combined behavioural and pharmacological protocol, including clomipramine (group C) and cats managed only with behavioural modification alone, without pharmacotherapy (group B). Baseline characteristics did not differ between groups in terms of age, sex, use of ultrasonography, and interval between onset of clinical signs and initiation of therapy, indicating that the groups were broadly comparable at the start of treatment. Despite this, cats receiving clomipramine showed a higher likelihood of positive outcome, a shorter time to recovery, and more consistent follow-up completion. These results suggest that in cases of unacceptable elimination exhibiting compulsive-like features, the inclusion of clomipramine in the therapeutic plan may confer a clinically relevant advantage, supporting a treatment approach aligned with a compulsive behaviour framework.

In clinical practice, elimination-related problems may be inadvertently normalised by both caregivers and general veterinary practitioners, particularly when the behaviour has been present for a long time and is perceived as ‘part of what cats do’ [33]. Conceptualising these presentations within a compulsive-like pattern may help shift the interpretation away from intentionality-based attributions (e.g., ‘spite’) or benign acceptance (‘we can live with it’) towards recognition of a pathological process that warrants active management [6].

The positive and favourable outcome of group C with respect to unacceptable urine deposition may thus be understood not merely as behavioural suppression, but as the result of an intervention targeting the underlying emotional and/or compulsive drivers of the behaviour [10]. Given that feline repetitive behaviours may function as displacement activities or coping strategies for frustration and fear, clomipramine’s efficacy might not be limited to compulsive patterns but may also extend to the regulation of the emotional distress driving these responses [10] Randomized controlled trials have already demonstrated significant reductions or the complete resolution of urine marking and spraying (but not specifically in unacceptable urination) in a substantial proportion of cats treated with clomipramine [34], without inducing generalised sedation, thereby reinforcing its role as an anxiolytic anti-compulsive agent [16]. However, it should be acknowledged that these trials primarily addressed territorial marking behaviours rather than elimination problems associated with primary environmental or psychosocial stressors, as in the present case series. Therefore, while these findings support the involvement of emotional dysregulation in certain forms of feline elimination behaviour, their direct applicability to our diagnostic category should be interpreted with caution.

Consistently, previous results have shown that adjunctive interventions (for example, pheromonatherapy and behavioural modification to remove any specific triggers) beyond general management advice significantly increase the probability that unacceptable urination reduced or ceased [34,35].

Many behavioural disorders in cats arise from disruptions to emotional equilibrium, stemming from perceived or actual threats or the frustration of thwarted natural behaviours [36]. Such emotional dysregulation may manifest as distress and behavioural pathology, with unacceptable urination representing prominent clinical expressions, alongside aggression and flight responses [37]. In the case of CD, these behaviours persisted even after potential environmental triggers have been eliminated or adequately managed [38]. This pattern is consistent with the conceptual framework whereby compulsive behaviours initially emerge in situations of frustration or conflict but subsequently become persistent and independent of the original eliciting context [38].

Taken together, our findings might support the hypothesis that unacceptable elimination shares core features with CD-like patterns [31]. All included cases fulfilled the fundamental characteristics of CD—namely, repetitiveness, redundancy, and rigidity—as reported by caregiver. At this stage of the discussion, it is crucial to account for both compulsive-like patterns and established behavioural patterns. The observed habitual responding—specifically the repetitiveness of daily elimination in a fixed location—frequently signals a consolidated substrate or location preference rather than a primary neurobiological deficit [39]. Once a feline identifies a specific site as safe or preferred, the behaviour becomes highly predictable [4]; this regularity does not necessarily imply a failure in the inhibitory control systems typical of CD [30].

Furthermore, the observed behavioural ‘‘rigidity,’’ characterized by persistent attempts to access the site, likely reflects high motivation or goal-directed behaviour, which is a hallmark of anxiety-based urine marking [40]. Unlike true compulsive behaviours, which are often ‘‘emancipated’ from their original functional goal, the intense drive to reach a specific location suggests that the context remains the primary driver. Consequently, these repetitive actions may also be interpreted as functional coping strategies or displacement activities, serving as stress-coping mechanisms in response to environmental stressors [10]. Further research should focus on these questions as well.

Moreover, the observed clinical response parallels that described in human OCD, in which anti-compulsive pharmacotherapy (e.g., SSRIs, selective serotonin reuptake inhibitors, clomipramine) is often required in addition to behavioural therapy to achieve remission and reduce the risk of relapse [41]. The cross-species consistency strengthens the argument that feline unacceptable elimination is not merely a problematic behaviour or an anxiety-driven elimination pattern [3], but rather a disorder that may lie on the compulsive spectrum.

The division into two treatment groups allowed us to compare the clinical course under behavioural management alone versus combined behavioural and pharmacological intervention. Cats in Group C (behavioural + pharmacological treatment) showed markedly superior outcomes compared to those in Group B (behavioural treatment only). The rapid clinical improvement observed within 30 days or less may be more consistent with a predominantly anxiolytic effect of clomipramine rather than a strictly anti-compulsive mechanism, as compulsive-like patterns are typically expected to show a more gradual response to treatment [42]. These findings indicate a marked difference in the temporal dynamics of recovery between groups; specifically, cats in Group C exhibited a swift and consistent resolution of symptoms, as reflected by the relatively short median time of 30 days. In contrast, in group B, a substantial proportion of subjects did not achieve recovery within the study period, resulting in an undefined median time. The significant log-rank test confirms that the probability of recovery over time differs between groups, suggesting a clear advantage of the condition represented by group C. Importantly, the absence of a measurable median in group B reflects the presence of censored observations and highlights the importance of using time-to-event methods to appropriately account for incomplete outcomes [43].

These findings indicate that pharmacological intervention targeting compulsive symptomatology may substantially accelerate recovery and promote long-term stability [9,16]. Clomipramine is a tricyclic antidepressant that inhibits the reuptake of serotonin and norepinephrine and also exerts anticholinergic, antihistaminic, and α_1_-adrenergic antagonist effects [15]. It has demonstrated efficacy in urine-related behavioural problems in cats and is commonly used in veterinary behavioural medicine [16].

Of additional interest in the context of unacceptable urination is its anticholinergic activity, which may reduce micturition frequency and therefore contribute to a reduction in the number of urination events in unacceptable locations [14]. However, clomipramine has also shown proven efficacy in the treatment of behaviours associated with anxiety OCD [16], emotional states that are frequently implicated in the aetiology and maintenance of unacceptable urination. The pharmacodynamic profile of clomipramine thus provides a plausible mechanistic framework for the improved outcomes observed in group C.

By recognising unacceptable urination as a compulsive analogue, clinicians may may be better equipped to approach treatment with greater precision, tailoring multimodal interventions that combine environmental modification with targeted pharmacotherapy, ultimately improving both feline welfare and caregiver quality of life.

The difference in time to recovery between the two treatment groups was substantial. Cats treated with clomipramine recovered within a relatively short period, whereas cats in the non-pharmacological group required considerably longer and some did not achieve full resolution within the observation period. From a clinical standpoint, a reduction in the duration of unacceptable urination episodes is relevant for feline welfare and for the preservation of the human–animal relationship, as elimination problems represent a frequent cause of relinquishment and euthanasia [44]. A shorter recovery time may therefore confer indirect benefits beyond symptom control, by decreasing the cumulative burden of the problem on caregivers and reducing the risk of permanent breakdown of the human–animal bond [29].

Follow-up completion was consistently higher in the clomipramine group. Several factors may contribute to this difference. It is possible that earlier and more marked clinical improvement have increased caregiver motivation to attend follow-up visits, thereby enhancing data completeness in group C. Conversely, slower or less responses in group B may have reduced adherence to scheduled re-evaluations. This discrepancy in follow-up could theoretically bias the estimation of long-term outcomes, particularly in the non-pharmacological group, in which late improvements or relapses might have been under-detected. Nevertheless, the magnitude and consistency of the differences observed in both outcome and time to recovery suggest that differential follow-up alone is unlikely to account for the overall pattern of results.

The findings of this study support the integration of pharmacological treatment into a multimodal management strategy for urine-related behavioural problems in cats [34]. In particular, clomipramine may help reduce underlying anxiety and compulsive components, thereby facilitating the implementation and effectiveness of environmental and behavioural interventions [9]. In clinical practice, its use should be combined with the optimisation of litter box conditions, environmental enrichment, and caregiver education, in line with current recommendations in veterinary behavioural medicine [5]. Such an integrated approach is likely to offer the greatest probability of sustained resolution [6]. The significance of this study lies in its potential to refine the diagnostic approach to feline unacceptable urination. Given that unacceptable urination is a primary driver for relinquishment and euthanasia, a more nuanced clinical assessment is vital for improving both feline welfare and owner compliance.

Accordingly, clomipramine represents a valuable therapeutic option in the management of unacceptable elimination in cats, particularly when urination appears to be driven by both anxiety and compulsive-like tendencies [16]. When integrated into a broader treatment plan that includes environmental and behavioural interventions, clomipramine may substantially improve clinical outcomes associated with unacceptable elimination.

Several limitations must be acknowledged. First, the sample size was relatively small, which limits statistical power and restricts the generalisability of the findings. Accordingly, these results should be interpreted with caution and do not allow definitive conclusions regarding the superiority of one therapeutic approach over another. The primary limitations of this research include its retrospective design and the reliance on owner-reported data. While providing valuable clinical insights, such an approach is inherently exploratory. Such studies are intended to generate hypotheses for future research rather than confirm definitive causal links. Consequently, a conclusive causal relationship cannot be established, particularly regarding the specific role of clomipramine in the observed clinical improvements. However, these present findings may provide a useful foundation for future prospective studies designed to further investigate treatment responses in cats with compulsive-like elimination patterns. Given the pharmacological profile of clomipramine, careful clinical monitoring (for example, blood and urine work) remains warranted, particularly in cats with concomitant medical conditions.

## 5. Conclusions

The primary objective of this study was to investigate long-standing cases of house-soiling to determine whether they exhibit clinical features consistent with a compulsive-like elimination pattern. Our findings demonstrate that cats treated with a combined behavioural and pharmacological protocol (Group C) experienced a higher likelihood of a positive outcome, a significantly shorter time to recovery, and higher follow-up completion rates compared to those managed with behavioural modification alone (Group B).

These results might suggest that when unacceptable urination displays compulsive-like characteristics, the integration of clomipramine into the therapeutic plan may offer a substantial clinical advantage. Such evidence supports the adoption of a treatment framework aligned with compulsive behaviour models for chronic cases. However, given the exploratory nature of this work, further research is warranted. Future prospective, randomized, and placebo-controlled trials—utilizing strict diagnostic differentiation between marking and toileting behaviours—are essential to validate these findings and refine the pharmacological management of feline unacceptable urination.

## Figures and Tables

**Table 1:** Demographic data on cats involved and details on recovery.

						Clinical Outcomes			
Group	Reproductive Status	Gender	Age (mo)	Time Between Onset of Clinical Signs and Start of Therapy (Days)	Time to Clinical Recovery (Days)	1 mo	3 mo	6 mo	1 y
						Outcome Positive = 1 Negative = 0			
C	N	F	36	365	30	1	1	1	1
C	N	F	36	365	30	1	1	1	1
C	N	M	60	605	20	1	1	1	1
C	N	M	60	605	20	1	1	1	1
C	N	F	96	455	40	0	1	1	1
C	N	F	60	365	30	1	1	1	1
C	N	M	64	365	20	1	1	1	1
C	N	M	13	425	60	0	1	1	1
C	N	M	90	400	20	1	1	1	1
C	N	M	48	425	20	1	1	1	1
C	N	M	24	365	30	1	1	1	1
B	N	M	65	365	50	0	1	1	1
B	N	M	15	365	30	1	1	1	1
B	N	F	72	720	120	0	0	1	1
B	N	M	13	1095	365	0	0	0	0
B	N	F	63	425	365	0	0	0	0
B	N	F	96	365	365	0	0	0	0
B	N	M	15	365	50	0	1	1	1
B	N	M	8	425	365	0	0	0	0
B	N	M	26	400	365	0	0	0	0
B	N	F	120	365	365	0	0	0	0

Gender: M = male; F = female; mo = months, y = years. Group: Clomipramine group (C), Behaviour-only group (B). Reproductive status: N: neutered. Time to clinical recovery (days): number of days from therapy initiation to complete resolution of unacceptable urination. Clinical outcomes: coded as positive (1) if the caregiver reported complete absence of unacceptable urination since the previous assessment, and negative (0) if unacceptable urination persisted or recurred during that interval.

**Table 2:** *p*-values from Fisher’s exact test for categorical variables and from the Mann–Whitney U test for time to recovery. Data are presented as median (interquartile range, IQR) or *n*/*N* (%).

Variable	Group B (*n* = 10)	Group C (*n* = 11)	*p*-Value
Positive outcome, *n*/*N* (%)	4/10 (40.0%)	11/11 (100.0%)	0.001
Follow-up at 1 month, *n*/*N* (%)	1/10 (10.0%)	10/11 (90.9%)	0.001
Follow-up at 3 months, *n*/*N* (%)	3/10 (30.0%)	11/11 (100.0%)	0.001
Follow-up at 6 months, *n*/*N* (%)	3/10 (30.0%)	11/11 (100.0%)	0.001
Follow-up at 1 year, *n*/*N* (%)	4/10 (40.0%)	11/11 (100.0%)	0.012

## Data Availability

The original contributions presented in this study are included in the article. Further inquiries can be directed to the corresponding author.
